# *Candida* spp. DNA Extraction in the Age of Molecular Diagnosis

**DOI:** 10.3390/microorganisms11040818

**Published:** 2023-03-23

**Authors:** Smaranda Ioana Codreanu, Cristina Nicoleta Ciurea

**Affiliations:** 1 Faculty of Medicine, “George Emil Palade” University of Medicine, Pharmacy, Sciences and Technology of Târgu Mures, 38 Gheorghe Marinescu Street, 540139 Târgu Mures, Romania; 2 Department of Microbiology, Faculty of Medicine, “George Emil Palade” University of Medicine, Pharmacy, Sciences and Technology of Târgu Mures, 38 Gheorghe Marinescu Street, 540139 Târgu Mures, Romania

**Keywords:** DNA extraction, candidemia, molecular diagnostics, PCR, Candida

## Abstract

The standard procedure for the detection of candidemia is blood culture, a method that might require 3–5 days for a positive result. Compared with culturing, molecular diagnosis techniques can provide faster diagnosis. The current paper aimed to present the main strengths and constraints of current molecular techniques for *Candida* spp. DNA extraction, analyzing their efficiency from a time, price, and ease of usage point of view. A comprehensive search was conducted using the PubMed NIH database for peer-reviewed full-text articles published before October 2022. The studies provided adequate data on the diagnosis of the infection with the *Candida* spp. DNA extraction is a relevant step in yielding pure qualitative DNA to be amplified in molecular diagnostic techniques. The most used fungal DNA extraction strategies are: mechanical (bead beating, ultrasonication, steel-bullet beating), enzymatic (proteinase K, lysozyme, lyticase), and chemical extraction (formic acid, liquid nitrogen, ammonium chloride). More clinical studies are needed to formulate adequate guidelines for fungal DNA extraction as the current paper highlighted discrepancies in the reported outcome.

## 1. Introduction

Once a rare and underdiagnosed infection, *Candida* spp. infections have taken more and more space in the last three decades. *Candida* spp. are among the top ten most frequent bloodstream pathogens and ranks as the number one cause of fungal infections, being able to cause superficial as well as deep infections [1,2,3,4].

Superficial infections (mucocutaneous candidiasis) can be either non-genital (e.g., oropharyngeal disease) or genitourinary (vulvovaginal candidiasis, balanitis, balanoposthitis) and they can occur on immunocompromised, as well as immunocompetent, individuals [5]. On the other hand, invasive candidiasis is associated with prolonged hospital stays and catheter use and in severe forms of septic shock, it can have a mortality of over 60% [6,7].

The population of neutropenic or immunocompromised patients is at the outermost risk of developing a form of fatal *Candida* spp. infection, and, as their number rises, so should our diagnostic tools [8,9,10,11,12,13]. The time of diagnosis is one of the most important predicting factors of mortality, as up to 50% of immunocompromised patients are diagnosed with a systemic mycosis post-mortem [14,15].

Fast identification of the pathogen allows the selection of the right treatment, ensuring better survival rates for the patients. A reliable method of diagnosis could ensure prompt time for starting the antifungal therapy and de-escalate if needed, decreasing multi-drug resistant pathogens [16,17,18,19]. Beginning an adequate empirical antibiotic treatment seems to be a variable that impacts independently the survival rates and prognosis of critically ill patients. Therefore, in an era where antibiotic stewardship is a widely debated topic, antifungal sensitivity and fungal speciation should be a priority.

The specific and time-efficient fungal diagnostic methods were not discussed widely enough in the current technological context. There is a stringent need for molecular diagnostics which translates into an increasing number of commercial extraction kits and in-house protocols that were developed over the last few years. A molecular diagnosis means faster results (less than 6 h), better selection of specific fungal therapy, and less biological material needed for correct identification compared to culture. Using polymerase chain reaction (PCR) methods reaches better and faster results than blood cultures (BC) and is a preferred approach in modern microbiology laboratories, as it proves to correctly diagnose up to 95% of invasive candidiasis cases [20,21]. The key to reaching good specificity and sensitivity is being able to extract high-quality, pure, fungal DNA from different samples (urine, blood, sputum, and swabs) [22]. That is the reason why establishing the highest-yielding methods should be a priority in the era of molecular diagnostic methods.

The current paper presents molecular techniques for *Candida* spp. DNA extraction and aims to highlight the advantages and disadvantages of each objectively. To determine the best approach based on the laboratory’s features, a comparative assessment is needed by identifying and describing different laboratory approaches: in-house protocols and commercial kits. The novelty of the review lies in comparing not only different approaches of the same method but also comparing different methods to each other.

## 2. Materials and Methods

A comprehensive search was conducted analyzing all research articles before October 2022 and included the PubMed NIH database. The guidelines for the selection and search were according to the PRISMA Checklist [23] and Cochrane Handbook for Systematic Reviews [24]: provide a clear objective of the review, present transparently the database interrogation criteria, present the results and limitations of the included studies, and discuss the limitations of the review and current practices.

The search was limited to English for accuracy reasons. Controlled vocabulary with keywords was used to search for studies of fungal DNA extraction. The keywords were ‘*Candida* spp. DNA extraction’ and ‘*Candida* molecular diagnosis’. Primers of any type and all targeted genes were accepted into the review.

The selection of the studies was carried on based on a two-step approach. The first step consisted of a preliminary assessment based on the title and the abstract, in order to eliminate ineligible results. The second step consisted in assessing the suitability of the papers, based on a full text read.

### 2.1. Inclusion Criteria

Included studies were peer-reviewed full-text articles that provided adequate data on the diagnosis of the infection with the *Candida* spp. The criteria proposed by the European Organization for Research and Treatment of Cancer/Invasive Fungal Infections Cooperative Group [25] and the Practice Guidelines of the American Thoracic Society [26] were followed. The recommendations included taking into consideration the DNA PCR methods as possible approaches for diagnosing invasive candidiasis, not using (1,3)-β-d-Glucan as a single method for diagnosing *Candida* spp. infections, and researching the impact of timely diagnosis on lowering mortality in patients with fungal infections.

### 2.2. Exclusion Criteria

The non-English papers and studies with fewer than 25 samples before enrollment in the study were excluded for feasibility reasons. The case reports were excluded as not being relevant for the review, as well as editorial letters and any paper published after October 2022.

## 3. Results

The initial database searches revealed 533 results, after the removal of the duplicates and the articles added from the reference list checking, 25 articles were left.

The included papers were 19 studies on yeast extraction protocols and six systematic reviews focusing on the guidelines for fungal diagnosis with biochemical and molecular techniques (to ensure adequate credibility, the AMSTAR tool [27] was used).

The identified methods presented below include the following extraction strategies: mechanical (bead beating, ultrasonication, steel-bullet beating), enzymatic (proteinase K, lysozyme, lyticase), and chemical (formic acid, liquid nitrogen, ammonium chloride).

The enzymatic strategies mentioned in short need a more detailed presentation as they play a key role in the extraction methods. Lysozyme is a glycolytic hydrolase that breaks the bond between N-acetylmuramic acid (NAM) and N-acetyl glucosamine (NAG), usually present in the cell wall of Gram-positive bacteria. Its addition to extraction methods seems to also improve the detection of *Candida* spp. DNA, although the fungus does not possess such bonds, the enzyme seems to act between the chitin substrate and the cell wall [28,29,30].

Lyticase (zymolase) is an enzyme complex that acts on the β(1→3)-glucose, an important component of the yeast cell wall [31,32,33]. Proteinase K is used to remove protein contamination and inactivate DNAases and RNAases that could potentially affect the sample. This endopeptidase is stable on a wide range of pH and temperatures and tends to work well on samples containing EDTA and calcium [31,34,35]. Regardless of the used enzyme, it is important to mention that there are reported cases of contaminated enzymes [36,37].

The lack of a gold standard when comparing methods and results for DNA extraction techniques makes it complicated for microbiologists to choose the best-suited option for their laboratories.

Table 1 and Table 2 summarize the 66 extraction methods including commercial and in-house protocols that make the object of the current paper, mentioning the pretreatment used (when needed) and the results stated as extracted DNA (quantitative results) or as detection limit (qualitative results) based on the results of each study analyzed. Table 1 presents the extraction kits that can be used from various sample types, while Table 2 characterizes the extraction kits approved for blood specimens.

As seen in Table 1 and Table 2., some of the sources cited the *Candida* spp. detection limit, some sources cited the *Candida* DNA quantity, and some delivered information about the total fungal or microbiological DNA without clear speciation. Extraction methods differed in terms of pretreatment, sample type, and detection limits, and might be difficult to compare, especially as there is no standardized method to express the sensitivities of different extraction kits. Moreover, some of the studies did not provide a clear methodology and delivered quantitative data by the increase compared to the chosen standard methods (e.g., a commercial kit). In addition to that, most available comparative studies used samples from healthy individuals and spiked them artificially by inoculating them with known quantities of pathogens and diluting them accordingly [43,44].

Some studies that included more species of the *Candida* spp. genus reported a marked heterogeneity among the outcome (e.g., for *C. tropicalis* only NucliSENS^TM^ easyMAG^TM^ yielded satisfactory results even for 10 CFU/mL). The reason behind this phenomenon may be the biofilm producing capacity of some of the species which might require a more aggressive approach in extracting the DNA due to the biofilm’s hydrophobicity [43,50].

It is worth highlighting that some methods (e.g., EZ1^TM^ DNA Tissue Kit) had vastly different reported detection limits based on pretreatment (chemical, thermal, or no added pretreatment). In addition to that, the quantity used for probe analysis seems to have impacted the results. A possible explanation could be that the methods impact the DNA strands and the amplification process is less effective [38,44].

For other methods, the pretreatment seems to either make little difference in the retrieved DNA quantity or lower it (e.g., Maxwell 16 Cell LEV DNA Purification Kit, Maxwell 16 Blood DNA Purification Kit, High Pure PCR Template Preparation). Therefore, in these cases, pretreatment not only has no added benefit but also adds unnecessary time to the process [44].

The fungal concentration in different types of *Candida* spp. infections (superficial of deep infections) is different; hence, this should also be kept in mind when choosing the extraction method.

When comparing the methods based on the type of sample used (e.g., QIAamp DNA mini kit), there is a stark difference in the initial sample quantity used and the cut-off for the detection limit. This finding suggests that the comparative approach should also be made with the same pretreatment on different sample types to generate the best possible strategies [41,42].

The sample volume appears to have made little difference in the case of Polaris enrichment, delivering the same results for 1 vs. 5 mL of initial sample volume. However, there were not many studies comparing the impact of the sample size in diagnosis [47].

Each type of pretreatment strategy and the advantage and limitations of the methods mentioned in Table 1 and Table 2 is presented in detail in the sections below.

### 3.1. Mechanical Extraction

The database search retrieved a multitude of mechanical extraction methods each with its advantages and limitations, as described in Table 3.

The bead beating and steel bullet beating protocols will be discussed at large in the review.

Ultrasonication is currently used only in laboratory settings for the extraction of bacterial pathogens. However, it is worth mentioning as it provides a future alternative for mechanical extraction protocols [32,51].

The high-speed cell disruption is a method not used at large for medical diagnostic purposes. It involves centrifugation and sedimentation and it offers the benefit of being fast and efficient (handling 12 samples in 1 h). However, it appears to generate a high lysis of the DNA [33].

#### 3.1.1. Bead Beating

The bead beating is a novel approach that is added to provide a 135-fold increase in the quantity of extracted fungal DNA and separate it from the human DNA, especially in whole blood by additional cell lysis. The method was added as a supplementary step to the already existing automatic kit EZ1 DNA Tissue-Kit (Qiagen, Hilden, Germany) [38].

The cell lysis is achieved through collision and physical contact between the beads and the cells, and the size of the beads, the sample texture, the types of cells to be lysed, as well as the bead milling method, might influence the results [34]. Beads can be made of glass, tungsten carbide, zirconium oxide, stainless steel, silica, ceramic [35,52], each material having its own characteristics. The beads material might influence the disruption energy that is applied to the target.

The bead beating was used before or after proteinase K digestion [53], liquid nitrogen maceration [54], or a combination of both in spiked whole blood or respiratory rinse. The protocol included using either a single ceramic bead or three beads (Precelys Lysing Kit CK 14_0.5 mL, VWR International GmbH, Darmstadt, Germany). The beads are added to the samples and shaken at high-speed to provide mechanical destruction of the cell wall. The resulting samples were diluted, plated on Sabouraud Dextrose GC Agar, and incubated for four days at 30 °C [38].

In bead beating protocols, the three beads approach provides better cell wall destruction and DNA release increasing it by up to 100-fold, independent of the proteinase K and liquid nitrogen use [51]. Although the DNA quantity seems to increase, the quality appears to suffer, with an impaired length of strands. This effect was previously described and it should be taken into consideration when choosing more strenuous bead-beating protocols [52,55,56,57].

However, a study mapping the urinary tract microbiome indicated that a bead beating with two silica beads (Biospec Products, Inc.) increased the detected fungal DNA only by 2–3-fold. Despite taking double the time when compared to commercial kits (150–180 min vs. 75–90 min), the mechanical addition seems nonetheless effective [28].

In newer methods such as metagenomic next-generation sequencing (mNGS), gentle DNA extraction is preferred. Hence, bead beating has a negative outcome in this case, causing a loss of short DNA sequences [58].

It is noteworthy that for the fungal pathogens with a larger genome, bead beating results in significantly better DNA extraction when compared to ultrasonication. Ultrasonication transfers three times more energy and should provide superior results at a first glance, but it does not manage complete cell disruption in laboratory conditions [51].

Laborious and cost-intensive bead beating methods (e.g., magnetic beads) tend to be less frequently used as they deliver worse qualitative and quantitative results when compared to accessibly methods such as phenol-chloroform extraction [59].

The frequency of bead beating can negatively affect the lengths of *Candida* DNA fragments in PBS suspensions, and triple bead beating might compensate the fragmentation of DNA [38].

#### 3.1.2. Steel-Bullet Beating

A more cost-effective alternative to bead beating could arise: steel-bullet beating [40]. This method proposes steel bullets that can be reused and sterilized, compared to single-use ceramic or glass beads. However, further studies are needed to assess the risk of probe contamination and the level of experience when using this method.

Motamedi et al. investigated three steel-bullet protocols [60,61,62,63,64,65] and compared their results to the glass beads protocol [38]. The details of each method are pictured below (Table 4).

Methods 1 and 3 only differ from a purification method point of view. The purification method appears to make little difference from a DNA extraction point of view, but the commercial kit took 60 min compared to 75 min for the phenol-chloroform purification [40].

The lysis buffer used was: 100 mM NaCl, 1 mM EDTA, 10 mM Tris–HCl, 2% Triton X100, and 0.5% SDS. The freezing step consisted of one-hour incubation at −80°C. The control method was carried out according to Scharf et al. [38].

The steel-bullets were prewashed with a lysis buffer and added together with the samples in a cylinder to perform the DNA extraction. After this step, each series of samples was purified according to the described protocol with either phenol-chloroform [66] or a commercial kit (Yekta Tajhiz Azma, Iran).

The method involving freezing yielded slightly higher DNA concentrations due to a more fragile cell wall but was more time-consuming and more expensive. Therefore, the steel-bullet method is valid also without it. 

Overall, the superior method time and money-wise was Method 3, incorporating the steel bullet, the lysis buffer, and the commercial kit, making it a suitable option to be further explored [40].

It is relevant to mention that the authors of the study used an ex vivo model for onychomycosis and that the results can be highly impacted based on preparation [40,55].

### 3.2. Thermal Extraction

Thermal extraction is mainly used as an addition to either enzymes or mechanical beating. The two preferred methods are either bringing the samples to boil or freezing them using liquid nitrogen [28,38,40,45,67]. Liquid nitrogen has an impact on costs (being expensive to buy and deposit), while also posing a chemical hazard.

While freezing alone does not seem to make a significant difference in DNA yield [40], combined with 10 min of boiling, it appears to be superior to commercial kits alone or commercial kits with an added enzymatic step [45].

However, high temperatures come across as offering high purity without the need for complicated protocols or expensive equipment [41,45]. A newly proposed method for DNA extraction is Chelex-100/boiling. Chelex-100 is a compound mainly used in forensic sciences for detecting blood or cells. By boiling the samples, the DNA is released and with the newly formed Chelex-100/magnesium ion complex, the DNA denaturation is reduced. Thus, the method offers similar DNA quality to the commercial kit. The added advantage is that it halves the preparation time (20 min vs. 40 min) before the amplification process [41].

High temperatures pose to be ideal for fungal DNA extraction from older samples such as formalin-fixed, paraffin-embedded tissue [67]. The extraction rates in these cases are often lower than from fresh tissues and can represent a challenge when trying to diagnose a cohort retrospectively [68].

### 3.3. Enzymatic Extraction

Thirty-six different enzymatic pretreatment protocols were analyzed as described in Table 1 and Table 2.

The conclusion was that some commercial kits (e.g., DNeasy Blood and Tissue, PureLink Genomic DNA Mini, High Pure PCR Template Preparation) exhibited the same results with or without pre-treatments, detecting the *Candida* spp. at concentrations from 10^6^ CFU/mL and that compared between each other, the commercial kits with an enzymatic step included offer better results than those without [42,43,44,66].

Regarding which enzyme proves to be more efficient, Ackmann et al. suggested that a step with both lysozyme and lyticase offers significantly better results than either single enzymatic addition [28].

### 3.4. Chemical Extraction

For protocols involving PCR amplification, phenol-chloroform, ammonium chloride [69], and TTE (Triton-Tris-EDTA) [70] are the in-house chemical methods taken into consideration for fragilizing the fungal wall and lysing human DNA and erythrocytes. The phenol-chloroform seems to yield better DNA extraction compared to both automated kits and bead beating probably due to the loss during silica column purification, respectively, due to mechanical disruption of the fungal DNA.

### 3.5. Manual vs. Automated Extraction Kits

The DNA purity obtained with different types of kits from different manufacturers is greatly variable and also improves or declines based on the pretreatment applied. However, there is an objective parameter that seems to differ: the time needed to process the samples. For the automatic kits, the time is around 40 min, whereas for the manual ones, it adds up to 2 h. Regardless of the pretreatment strategies, comparing three automated kits to six manual ones revealed that they offer a comparative yield rate of the *Candida* spp. DNA [44].

As for which commercially available kits seem to perform better, the opinions are split based on adding supplementary extraction steps or following only the recommended protocols [69].

## 4. Discussion

The standard procedure for the detection of candidemia is blood culture (BC) [71,72], a method that needs between 3 and 5 days for a positive result. BC might also provide false negative results in proven cases of candidiasis, as only 8–32% of the patients with autopsy-verified invasive candidiasis were diagnosed antemortem [14]. The collection methods highly influence the BC results, as BC might become easily contaminated, or improper collection techniques might affect the viability of microorganisms. Using blood or serum samples to search directly for the pathogenic antigens is a nonculture method that was discussed, but was proven to offer low sensitivity [73,74,75].

The importance of molecular diagnosis relies on the fact that conventional diagnosis (culturing) might provide late or even false negative results in some cases. Non-culture diagnosis methods (e.g., antibody, antigens, polymerase chain reaction) are currently part of the medical practice, as complementary tests, next to traditional culturing [76]. The purification of DNA stands at the basis of molecular analysis.

Compared with bacterial DNA, fungal DNA extraction protocols poses challenges, mostly regarding the toughness of the fungal cell walls [77]. Successful extraction of the fungal DNA means understanding the particularities of the *Candida* spp.’s cell walls containing 1,3-beta-D-glucan (BDG), chitin, and mannan. The three main components can be used to detect the fungal levels in blood samples but are not specific for yeast infections. Additionally, some species tend to produce biofilms, making them harder to diagnose and treat. The special cell wall composition makes *Candida* spp. detection a difficult process, requiring high temperatures or toxic agents to extract the genomic DNA [78,79,80,81,82].

In the case of critically ill patients, it is worth searching for a method that would be independent of the biological sample (blood, sputum, saliva, oral rinse) and in small sample volumes for frequent testing to perform the extraction at a satisfactory level. As such, the fungal infection might have a different load and, therefore, be harder to diagnose based on its site (e.g., *C. albicans* is easier to detect in serum when compared with whole blood and sputum presents commensal pathogens that are not the cause of the infection) [19,83,84].

In many cases, candidiasis in different patient populations is species-dependent, with *C. albicans* being the most common strain, *C. glabrata* being more prevalent in organ-transplanted patients, *C. parapsilosis* and *C. tropicalis* being more present in the southern hemisphere and *C. krusei* targeting patients with hematological malignancies [4,23,24]. These strains tend to make up for more than half of the diagnosed candidiasis in hospital settings, but their prevalence varies greatly based on geographical area [85,86,87,88]. A misdiagnosis usually occurs because of the mix of species in the sample or because of the close phenotypic profile [89].

The right treatment makes an impact on candidemia, as the mortality of the patients diagnosed in the first 24 h after the onset of the disease was 15.40% versus over 40% when diagnosed later than 72 h [90]. The timely start of antifungal therapy also appears to have an impact on the hospital stay and a correct diagnosis might release a part of the financial burden of healthcare [91].

Moreover, several species (e.g., *Candida auris*) rose in numbers as nosocomial infections with high rates of mortality and the behavior of a multi-drug resistant fungus. The current methods are inefficient in detecting the pathogen at low concentrations, and that is the reason why a comparative approach of the different methods could make a great difference in these cases and could potentially prevent fungal outbreaks [83,84,92,93,94].

One of the main limitations of the current review was that the samples came from a mock population consisting of biological samples from healthy patients and inoculated with fungal cells. In the cited literature, there is a limited number of studies using a sick population for systemic candidiasis. Löffler et al. reviewed the samples from neutropenic patients suspected with systemic candidiasis [48]. Richard et al. also proposed an efficient method of DNA extraction from a clinical population, but there were only two patients in the cohort and the results were NOT tested further [95].

## 5. Conclusions

The current methods for detecting *Candida* spp. at low concentrations are highly dependable on the extraction protocols used. Thus, a comparative approach of the different methods could make a great difference and could potentially prevent fungal outbreaks and reduce mortality. Naturally, it is to be considered that each reported result is also influenced by the amplification method that was chosen.

A wide variety of commercial kits also translates in applicability to different types of biological samples having different concentrations of human DNA. Therefore, validation in clinical settings is needed before choosing the best extraction method.

## Figures and Tables

**Table 1:** Characteristics of the analyzed extraction kits and applied pretreatments for various samples types of superficial *Candida* spp. infections [28,38,39,40,41,42].

Kit’s Description	Manufacturer	Sample Type	Sample Volume	Pretreatment Method	Extracted DNA (ng/μL)	Species	Reference
Automatic kits							
IndiSpin Pathogen Kit	Indical Bioscience	inoculated urine *	200 μL	-	22.8	*C. albicans*	[19]
IndiSpin Pathogen Kit	Indical Bioscience	inoculated urine *	200 μL	glass beads	9.2	*C. albicans*	[19]
IndiSpin Pathogen Kit	Indical Bioscience	inoculated urine *	200 μL	LB	39.2	*C. albicans*	[19]
Manual kits							
In-house protocol		urine **	1 mL	LyS	6	*Candida* spp.	[27]
In-house protocol		urine **	1 mL	LyT	8	*Candida* spp.	[27]
In-house protocol		urine **	1 mL	LyS LyT	14	*Candida* spp.	[27]
In-house protocol		inoculated hooves ***	20 mg	steel bullet LB phenol chloroform	244 ± 31.27	*C. albicans*	[40]
In-house protocol		inoculated hooves ***	20 mg	freezing steel bullet LB phenol chloroform	366 ± 49.69	*C. albicans*	[40]
In-house protocol		inoculated hooves ***	20 mg	steel bullet LB commercial kit	169.2 ± 27.94	*C. albicans*	[40]
In-house protocol		inoculated hooves ***	20 mg	bead beating LB phenol chloroform	117 ± 32.48	*C. albicans*	[40]
QIAamp DNA mini kit	Qiagen	mouth rinse ****	1 mL	-	30	*Candida* spp.	[42]
QIAamp DNA mini kit	Qiagen	mouth rinse ****	10 µL	LyT PK	15	*Candida* spp.	[42]
QIAamp DNA mini kit	Qiagen	mouth rinse ****	10 µL	LyT PK silica beads	10	*Candida* spp.	[42]
In-house protocol		mouth rinse ****	10 µL	PK SDS phenol chloroform	18	*Candida* spp.	[42]

LyS = lysozyme, LyT = lyticase, PK = proteinase K, LB = lysis buffer, SDS = sodium dodecyl sulphate. Sample preparation: * urine samples from patients presenting clinically with symptomatic UTI, ** standardized mid-stream urine samples from healthy male and female participants,*** inoculated hooves as ex vivo onychomycosis model, **** mouth rinse from healthy patients

**Table 2:** Characteristics of the analyzed extraction kits on blood specimens and applied pretreatments as DNA extraction strategies.

Kit’s Description	Manufacturer	Whole Blood Specimens Volume	Pretreatment Method	Detection Limit	Species	Reference
Automatic kits						
EZ1^TM^ DNA Tissue Kit *	Qiagen	200 μL	-	1400 ng/μL	*C.glabrata*	[38]
EZ1^TM^ DNA Tissue Kit *	Qiagen	200 μL	N2	570 ng/μL	*C.glabrata*	[38]
NucliSENSTM EasyMAGTM *	Fisher Scientific	200 µL	CB LB	10 CFU/mL Ct < 20	*C. albicans* *C.glabrata* *C.parapsilosis* *C.tropicalis* *C. krusei*	[43]
EZ1^TM^ DNA Blood 200 µL Kit *	Qiagen	200 µL	-	10^6^ CFU/mL		[43]
EZ1^TM^ DNA Blood 200 µL Kit *	Qiagen	200 µL	CB LB	10 CFU/mL Ct < 20		[43]
EZ1^TM^ DNA Tissue Kit *	Qiagen	190 µL	LyT	10 CFU/mL Ct < 20		[43]
EZ1^TM^ DNA Tissue Kit *	Qiagen	100μL^−1^ mL	-	10^3^ CFU/mL	*C. albicans*	[44]
EZ1^TM^ DNA Tissue Kit *	Qiagen	100μL^−1^ mL	TTE LyS LyT	10^2^ CFU/mL	*C. albicans*	[44]
QIAamp^TM^ 96DNA QIAcube HT kit *	Qiagen	200 μL	-	10^6^ CFU/mL	*C. albicans* *C.glabrata* *C.parapsilosis* *C.tropicalis* *C. krusei*	[43]
Macherey-Nagel™ Pathogène NucleoMag™ *	BioMérieux	150 μL	-	10^6^ CFU/mL		[43]
Mag-Bind^TM^ Viral DNA/RNA kit *	Omega Bio-tek	200 μL	-	10^6^ CFU/mL		[43]
MagMAX™ Viral/ PathogenNucleic Acid Isolation Kit *	Applied Biosystems MGISP	400 μL	-	10^6^ CFU/mL		[43]
Chemagic Viral DNA/RNA 300 kit H96 *	PerkinElmer	200 μL	-	10^6^ CFU/mL		[43]
Virus DNA/RNA Extraction Kit *	MGI	200 μL	-	10^6^ CFU/mL		[43]
Bioextract^TM^ Superball^TM^ kit *	Biosellal	200 μL	-	10^6^ CFU/mL		[43]
Maxwell 16 Cell LEV DNA Purification Kit *	Promega Co.	100μL^−1^ mL	-	10^2^ CFU/mL	*C. albicans*	[44]
Maxwell 16 Cell LEV DNA Purification Kit *	Promega Co.	100μL^−1^ mL	TTE LyS LyT	10^2^ CFU/mL	*C. albicans*	[44]
Maxwell 16 Blood DNA Purification Kit *	Promega Co.	100μL^−1^ mL	-	10^6^ CFU/mL	*C. albicans*	[44]
Maxwell 16 Blood DNA Purification Kit *	Promega Co.	100μL^−1^ mL	TTE LyS LyT	10^2^ CFU/mL	*C. albicans*	[44]
Manual kits						
DNeasy Blood and Tissue *	Qiagen	100μL^−1^ mL	-	Not detected	*C. albicans*	[44]
DNeasy Blood and Tissue *	Qiagen	100μL^−1^ mL	TTE Ammonium chloride LyS LyT Bead Beating	10^2^ CFU/mL	*C. albicans*	[44]
QIAamp DNA Blood Min i *	Qiagen	100μL^−1^ mL	-	10^6^ CFU/mL	*C. albicans*	[44]
QIAamp DNA Blood Mini *	Qiagen	100μL^−1^ mL	LyS LyT Bead Beating	10^2^ CFU/mL	*C. albicans*	[44]
PureLink Genomic DNA Mini *	Invitrogen Co	100μL^−1^ mL	-	10^6^ CFU/mL	*C. albicans*	[44]
PureLink Genomic DNA Mini *	Invitrogen Co	100μL^−1^ mL	LyS LyT	10^6^ CFU/mL	*C. albicans*	[44]
High Pure PCR Template Preparation *	Roche Inc.	100μL^−1^ mL	-	10^6^ CFU/mL	*C. albicans*	[44]
High Pure PCR Template Preparation *	Roche Inc.	100μL^−1^ mL	LyS LyT	10^3^ CFU/mL	*C. albicans*	[44]
UMD-Universal CE IVD *	Molzym GmbH & Co.	100μL^−1^ mL	-	10^1^ CFU/mL	*C. albicans*	[44]
QIAamp DNA mini kit *	Qiagen	2 µL	-	< 10 ng/μL	*Candida spp.:* *C. albicans* *C. glabrata* *C. parapsilosis* *C. tropicalis* *C. famata* *C. krusei* *C. dubliniensis* *C. haemulonii*	[41]
QIAamp DNA mini kit *	Qiagen	2 µL	SDS β-mercaptoethanol	20 ng/μL		[41]
QIAamp DNA mini kit *	Qiagen	2 µL	glass beads	198 ± 18.9 ng/μL		[41]
Chelex-100/boiling	Not commercial	200 μL	-	10^4^ CFU/mL	*C. albicans*	[45]
In-house protocol *	Not commercial	50–100 µL	guanidinium thiocyanate acid PK	260 CFU/mL (whole blood) 200 CFU/mL (serum)	*C. albicans*	[46]
QIAamp DNA Blood Mini *	Qiagen	1 mL	Polaris (Biocartis) enrichement LB	1 CFU/mL Ct < 35	*C. albicans*	[47]
QIAamp DNA Blood Mini *	Qiagen	5 mL	Polaris (Biocartis) enrichement LB	1 CFU/mL Ct < 35	*C. albicans*	[47]
In-house protocol **	-	3 mL	TTE SDS potasium acetate centrifugation cold ethanol	1–10 CFU/mL	*C. albicans*	[48]
GeneReleaser **	Eurogentec	3 mL	LB SDS TTE β-mercaptoethanol	1–10 CFU/mL	*C. albicans*	[48]
QIAamp Tissue **	Qiagen	3 mL	LB SDS TTE β-mercaptoethanol	10 CFU/mL	*C. albicans*	[48]
PureGene D 6000 **	Gentra	3 mL	LB SDS TTE β-mercaptoethanol	10^2^ CFU/mL	*C. albicans*	[48]
DNAzol **	Sigma	3 mL	LB SDS TTE β-mercaptoethanol	10^3^ CFU/mL	*C. albicans*	[48]
PKPC	Not commercial	400 μL	PK PC TTE	10^3^ CFU/mL	*Candida spp.*	[49]
HLGT	Not commercial	400 μL	guanidine thiocyanate acid heat lysis	10 CFU/mL	*Candida spp.*	[49]
QIAamp DNA Blood *	Qiagen	400 μL	-	10 CFU/mL	*Candida spp.*	[49]
High Pure PCR Template Preparation *	Roche Inc.	400 μL	-	10^2^ CFU/mL	*Candida spp.*	[49]
DNAzol *	Sigma	400 μL	-	10^4^ CFU/mL	*Candida spp.*	[49]
QIAamp DNA mini kit *	Qiagen	1 mL	LyT	96 ± 21	*C. albicans*	[31]
QIAamp DNA mini kit *	Qiagen	1 mL	glass beads	89 ± 44	*C. albicans*	[31]
MasterPure yeast DNA purification kit *	Epicentre	1 mL	TTE	215 ± 109	*C. albicans*	[31]
BAGH *	Not commercial	1 mL	benzyl alcohol guanidine hydrochloride TTE	33 ± 42	*C. albicans*	[31]
Dr GenTle (gene trapping by liquid extraction) *	Takara Bio	1 mL	TTE	36 ± 18	*C. albicans*	[31]
yeast DNA extraction reagent (Y-DER) *	Pierce Biotechnology	1 mL	TTE	23 ± 10	*C. albicans*	[31]
YeaStar genomic DNA kit *	Zymo Research	1 mL	TTE	11 ± 4	*C. albicans*	[31]

TTE = Triton-Tris-EDTA (20 mM Tris-HCl [pH 8.3], 1 mM EDTA, and 1% Triton), PK = proteinase K, LyS = lysozyme, LyT = lyticase, CB = ceramic beads, LB = lysis buffer, N2= liquid nitrogen maceration. * the study involved blood samples from healthy patients spiked with fungal cells, ** samples from neutropenic patients suspected or diagnosed with systemic candidiasis.

**Table 3:** Proposed methods for mechanical extraction [32,38,40,51].

Methods	Advantages	Limitations
Bead Beating	▪ significantly increases the extracted fungal DNA	▪ single use beads could prove to be expensive
Ultrasonication	▪ transfers more energy	▪ does not manage to lyse the cell wall in laboratory conditions
Steel Bullet Beating	▪ the bullets can be sterilized ▪ the method proves to be more efficient than the bead beating	▪ risk of contamination
High-Speed Cell Disruption	▪ the method proves to be as efficient as PC extraction ▪ cost and time-efficient	▪ shearing DNA

**Table 4:** Steel-bullet protocols adapted from *Motamedi* et al. [40].

Method no.	Extraction Method	Purification Method	Extraction Time (min)	Detection Limit Extracted DNA (ng/μL)
1	Steel bullet + lysis buffer	phenol-chloroform	5	244 ± 31.27
2	Freezing + Steel bullet + lysis buffer	phenol-chloroform	60	366 ± 49.69
3	Steel bullet + lysis buffer	commercial kit (Yekta Tajhiz Azma, Iran)	5	169.2 ± 27.94
Control	Bead beating + lysis buffer	phenol-chloroform	5	117 ± 32.48

## Data Availability

Not applicable.
